# The impacts of family planning and HIV service integration on contraceptive prevalence among HIV positive women in Tanzania: a comparative analysis from the 2016/17 Tanzania HIV impact survey

**DOI:** 10.1186/s40834-023-00260-w

**Published:** 2023-12-06

**Authors:** Saitoti Timoth, Jane Machange, Kilaye Karino, Sally Mtenga, Abdallah Mkopi, Francis Levira

**Affiliations:** 1 School of Life Sciences and Bioengineering, The Nelson Mandela African Institution of Science and Technology, P.O. Box 447, Arusha, Tanzania; 2 School of Diagnostic Medicine, Muhimbili University of Health and Allied Sciences, Dar es Salaam, Tanzania; 3 Department of Health Systems, Impact Evaluation and Policy, Ifakara Health Institute, #5 Ifakara Street, Mikocheni, P.O. Box 78 373, Dar es Salaam, Tanzania; 4 Kilimanjaro Christian Medical University College, P. O. Box 2240, Moshi, Tanzania

**Keywords:** Reproductive women, HIV positive, HIV negative, Modern contraceptive use, Tanzania

## Abstract

**Background:**

Prevention of unplanned pregnancies through modern contraceptives among HIV-positive women is one of the essential strategies for reducing mother-to-child transmission of HIV. Family planning and HIV services integration is a national strategy designed to scale-up modern contraceptives among HIV-positive women. This study aims to evaluate the success of a service integration strategy by comparing the prevalence of modern contraceptive use among HIV-positive women receiving ART within integrated services and those not on integrated services (HIV-negative women and HIV-positive women unaware of their status).

**Methods:**

We used data from the Tanzania HIV impact survey (THIS) of 2016/17. THIS provided HIV counselling and testing with a return of results in over 30,000 adults over 15 years of age. Women tested positive self reported their enrollment into ARV with further confirmation through laboratory analysis for any detectible ARV in their blood. All non-pregnant women reported their contraceptive use. Univariate and multivariate logistic regression was used to assess the effect of accessing integrated services controlling for potential confounders.

**Results:**

A total of 14,986 women were included in the analysis; HIV-positive women were 1,066 and HIV-negative women were 13,830. Modern contraceptive use prevalence was 35% among HIV-positive women and 30% among HIV-negative women. Among HIV-positive women, those enrolled in integrated services (ART) had a higher prevalence of modern contraceptive (40%) compared to HIV-positive women unaware of their status (27%, *p*-value = 0.0014). The most common contraceptive methods in HIV-positive women were injectables (32%) and male condoms (31%), while in HIV-negative women, injectables (39%) and implants (30%, *n* = 1032) were the most preferred methods. Among HIV-positive women, enrolment into integrated services (currently on ART) demonstrated an increase in the odds of modern contraceptives by 85% (AOD = 1.85, 95%CI: 1.27–2.71).

**Conclusion:**

This study found relatively low modern contraceptive use among HIV-positive women in the general population despite the existance of service integration program and guidelines to guide its implementation.Our study therefore calls for the evaluation on the implementation of the integration programme to identify factors that constrain or facilitate programme effectiveness.

## Introduction

Women and their reproductive actions are essential for the success of HIV prevention and control programs. Over half of 38 million people infected with HIV/AIDS globally are women [1]. Women of reproductive age (15–49) account for almost 40% of the global HIV burden [2]. There are 1.7 million children globally infected with HIV [1], with sub-Saharan African countries accounting for 90% of HIV-positive children globally [3]. Most of the transmission (90%) occurs through mother-to-child transmission (MTCT) [3]. Nearly two million HIV-positive women globally become pregnant with a high risk of passing HIV to their newborns due to unsafe sexual practices and limited access to modern contraceptives [4, 5]. Modern contraceptives are cost-effective in preventing MTCT of HIV [6, 7]. Besides reducing MTCT of HIV, contraceptives reduce pregnancy-related complications among WLWH, thus increasing the quality of life and time for engaging in education and economic activities [7]. Despite these benefits, the uptake of modern contraceptives among women living with HIV (WLWH) remains low, particularly in low- and middle-income countries (LMICs), as evidenced by high rates of unplanned pregnancies, abortions, and MTCT of HIV [8].

National governments and international organizations endorsed integrating family planning and HIV services as a strategy to reduce unintended pregnancies and MTCT of HIV [9]. Quasi- and randomized-experimental studies have demonstrated the positive impact of services integration on the uptake of modern contraceptives [10]. Tanzania developed National Operational Guidelines for maternal, newborn, child health (MNCH) and HIV/AIDS Integration (NOGI) strategy to address the MNCH and HIV challenges, including low modern contraceptive prevalence, high rates of unintentional pregnancy, and high MTCT [11]. NOGI provides a guide on family planning and HIV services integration for all levels of health service deliveries (dispensary, health facility and hospitals). Family planning and HIV treatment guidelines were updated to accommodate service integration needs as specified in NOGI [12, 13]. The current guidelines stipulate that healthcare providers at care and treatment centres (CTCs) should assess the needs for family planning for all its clients, including those on ART.Furthermore, the guideline recommends that, all individuals should be initiated with ant-retroviral therapy (ART) within seven days of a HIV positive diagnosis [12].

Tanzania endorsed the strategy and is implementing family planning and HIV services integration at all facility levels. During routine HIV care and PMTCT visits, all women of reproductive age should be assessed for pregnancy status and family planning needs, to facilitate FP counseling and provision of contraceptives or referrals.

Over 90% of HIV-positive women access ART through HIV treatment and PMTCT clinics [13]. HIV-positive women on ART represent women potentially benefiting from service integration in Tanzania. In a well-functional integrated program, HIV-positive women are expected to have higher modern contraceptive prevalence than other groups due to intensified family planning counselling, prescriptions and referrals [14, 15].

Using data from a large household-based HIV impact survey in Tanzania, we tested this hypothesis by comparing modern contraceptive use among women on ART (integrated services) with HIV-positive women not on treatment and HIV-negative women. This assessment will provide information on the performance of the service integration strategy in Tanzania and the need for its evaluation. While few facility-based surveys have examined the prevalence of modern contraceptives among HIV-positive women, to our knowledge, there is no national survey analysis on this subject in Tanzania.

## Materials and methods

### Study area

The United Republic of Tanzania comprises Tanzania mainland and Zanzibar Islands. The total population in 2022 was estimated at 59.8 million, where 69% of the people live in rural areas [16]. The average population growth rate is 3.2%, primarily driven by high fertility and low mortality rates [17]. The total fertility rate in the country is 4.8 per woman [18].

Tanzania is among the countries with the highest levels of HIV and unmet need for modern contraception in sub-Saharan Africa. Current modern contraceptive prevalence among married women stands at 31% [19]. Nearly one-third, or more than 600,000 births/pregnancies, are unwanted/mistimed annually [20]. The National HIV prevalence for adults (15–49) is 4.7% (6.0% in females and 3.3% in males) [13]. HIV prevalence in infants younger than 18 months born to HIV-positive females is 10.5% [13].

### Study design

This study utilized data from the Tanzania HIV impact survey (THIS) 2016/2017. THIS is a nationally representative household-based cross-sectional survey covering Tanzania's Mainland and Zanzibar Islands [13]. THIS was designed to estimate HIV prevalence and incidence, assess the impact of HIV care and treatment services, and characterize HIV-related risk factors. THIS offered home-based counselling and collected blood samples for testing and diagnosis of HIV-1, Hepatitis B, syphilis and hepatitis C from adults aged 15 years.

THIS employed a two-stage stratified cluster sampling design, with the first stage involving sampling enumeration areas (EA) and the second stage sampling households. Sampling was stratified by residency (urban and rural) and 31 geographical regions. The sampling frame for THIS survey was the 2012 Tanzania population and housing census survey. Sampling weights were generated and were used to account for survey design, selection probabilities and non-responses in statistical analysis.

### Data collection

THIS used adult, adolescent, and household questionnaires to collect social, demographic and household information. Our analysis focuses on data collected from household and adult questionnaires from households with women of reproductive age. The household questionnaire collected various members' data, including basic social and demographic information. The adult questionnaire was completed for one household member to capture, among others, sexual activity, pregnancy, and childbearing information and service utilization data, including family planning, ANC, and self-reported HIV status. Non-pregnant women responded to the contraceptive access and use modules. Data is achieved by the Population-Based HIV Impact Assessment (PHIA) project. We accessed the data from the project’s portal at https://phia.icap.columbia.edu/.

### Outcome variables

Modern contraceptive use among women of reproductive age is the primary outcome of this analysis. Non-pregnant women were asked whether “you or your partner are currently doing something or using any method to delay or avoid getting pregnant”. Those who responded yes were further asked to report their contraceptive method. Contraceptive methods were divided into modern methods (sterilization, condoms, intra-uterine devices, implants, pills, diaphragm, and injectables), traditional methods (withdrawal and rhythm) and non-use.

### Predictor variables

This study's primary predictor of modern contraceptives is the enrollment into integrated family planning and HIV service. HIV treatment guidelines require healthcare providers to offer family planning services when providing HIV treatment. Therefore, in this study, enrollment in HIV treatment is the primary predictor variable of interest. Enrolment in ART treatment was identified by asking HIV-positive respondents whether they were on ART treatment. Blood samples of all HIV-positive women were further screened for detectable levels of ARVs to confirm self-reported responses. The final service integration variable is binary indicators 1 if the respondent has detectable ARVs and 0 otherwise. Confounding predictor variables of interest were identified and grouped into social-demographic, reproductive, and HIV-related characteristics. Social-demographic variables collected by THIS include age, marital status, education level, place of residency (urban/rural), wealth quintiles, and occupation status, among many others. Reproductive characteristics included the number of pregnancies, children, births, and sexual partners. HIV-related characteristics included awareness of HIV status (partners status and self-status), duration of ARV treatment and suppression.

### Data analysis

We used descriptive statistics (Mean, standard deviations and percentages) to show the distribution of respondents' social-demographic, reproductive and HIV-related characteristics. Modern contraceptive prevalence was calculated by dividing the number of non-pregnant women who reported using any modern contraceptive (numerator) and the total number of non-pregnant women (denominator). The Pearson chi-square test was used to assess the differences in modern contraceptive use between the study groups. The univariate logistic regression model was used to assess the strength of the association between modern contraceptive use and one potential predictor at a time. We re-assessed the strength of association using a multivariate logistic regression model, which simultaneously accounts for multiple predictors in a single model. In multivariate analysis, we only included predictors with a p-value equal or less than 0.25 in univariate analysis. Sampling weight was applied to descriptive and regression analyses to account for survey design, selection probabilities and response rates. Odds ratios with 95% confidence intervals were computed to assess the strength of associations. Statistical significance was considered at a *p*-value less than 0.05. The data were analyzed using STATA version 15.

## Results

### Social-demographic characteristics of study participants

The final analysis includes 14,896 women; 1066 were HIV-positive, and 13,830 were HIV-negative. Among those diagnosed with HIV, 61% self-reported being aware of their HIV status. After combining self-report and ART detection in blood, 65% of HIV-positive women were estimated to be aware of their HIV status. Over 90% of women diagnosed with HIV are on ART. Viral suppression had been attained in most women on HIV treatment (79%) compared to those not on HIV treatment (20%).

Age distribution substantially differed among HIV-positive and negative women. Over 50% of HIV-positive women were older adults (35–49), while the majority of HIV-negative women (44%) were youth (15–24). Other characteristics differentiating the two groups were attainment of secondary education and above (HIV-positive = 13% vs HIV negative = 26%), living in urban areas (HIV-positive = 50% vs HIV-negative = 39%), and divorced/widow/separated marriage status (HIV-positive = 35% vs HIV-negative = 12%). Table 1 presents detailed social-demographic information on the study participants.

**Table 1:** Social-demographic characteristics of study participants

**Characteristic**	**HIV-positive (1,066)**	**HIV-negative (13,830)**	*P* value
	n (%)	n (%)	
**Age categories**			
15–24	146 (14.6)	5803 (44.0)	
25–34	379 (34.2)	4283 (29.9)	
35–49	541 (51.2)	3744 (26.1)	< 0.001
**Education Level**			
No formal education	199 (17.3)	2286 (14.3)	
Primary education	745 (70.1)	8255 (59.9)	
Secondary +	120 (12.6)	3282 (25.8)	< 0.001
**Residence**			
Urban	479 (49.9)	4762 (38.8)	
Rural	587 (50.1)	9068 (61.2)	< 0.001
**Currently working**			
Yes	233 (21.6)	2126 (15.6)	
No	833 (78.4)	11,688 (84.4)	< 0.001
**Household wealth**			
Low	634 (54.4)	8684 (58.0)	
High	432 (45.6)	5140 (42.0)	0.071
**Marital status**			
Never married	121 (13.0)	3458 (28.6)	
Married	564 (52.0)	8719 (59.7)	
Divorced/Widow/Sep	380 (35.0)	1626 (11.7)	< 0.001

### Sexual, reproductive and HIV-related characteristics of study participants (14,896)

There was a slight difference in the proportion of women who were sexually active in the past 12 months (HIV-positive = 77% vs HIV-negative = 84%). Multiple sexual partnerships in the past 12 months were more prevalent among HIV-positive (16%) than HIV-negative (9%). On the other hand, more HIV-positive women (34%) frequently reported condom use during non-marital sexual intercourse than HIV-negative women (26%). Pregnancies are fewer among HIV-positive (7%) than HIV-negative (12%) at the time of the survey. Birth histories in the past five years indicate fewer HIV-positive women (8%) gave birth to more than two children than HIV-negative women (14%). Table 2 provides women's detailed sexual and reproductive characteristics, and Appendix 1 summarizes the sexual characteristics of respondents.

**Table 2:** Sexual and reproductive characteristics among HIV-positive and negative women (*n* = 14,896)

Characteristic	HIV-positive	HIV-negative	*P* value
	**n(%)**	**n(%)**	
**Currently sexually active**			
Yes	807 (77.1)	10,040 (83.9)	
No	232 (22.9)	1939 (16.1)	< 0.001
**Relationship with last sex partner in the past 12 months**			
Spouse/live-in partner	415 (59.5)	6769 (70.8)	
Non-cohabiting	277 (40.5)	2423 (29.2)	< 0.001
**Number of sexual partners in the past 12 months**			
None	232 (23.0)	1939 (16.3)	
Only one	641 (60.5)	8887 (74.3)	
More than one	162 (16.5)	1026 (9.4)	< 0.001
**Condom use at last sexual intercourse with a non-cohabiting partner in the past 12 months prior to survey**			
Yes	121 (33.9)	798 (26.4)	
No	214 (66.1)	2112 (73.6)	< 0.001
**Current pregnancy status**			
Pregnant	76 (7.1)	1248 (11.6)	
Not pregnant	914 (92.9)	9411 (88.4)	< 0.001
**Number of pregnancies, including a current pregnancy**			
None	65 (7.1)	2939 (24.1)	
1–2	322 (32.5)	4066 (30.3)	
3 +	676 (60.4)	6720 (45.6)	< 0.001
**Number of children (past 5 years)**			
0	517 (51.6)	3173 (24.2)	
1	336 (33.1)	5032 (36.9)	
2 +	90 (7.8)	1951 (13.6)	
Never	65 (7.6)	2939 (25.3)	< 0.001

### Prevalence of modern contraceptive use and method preference among HIV-positive and negative women (*n* = 13,293)

Modern contraceptive prevalence among HIV-positive women was 35%, slightly higher than in HIV-negative women (30%) (*p*-value = 0.004)**.** In women diagnosed with HIV, the contraceptive prevalence was higher among women who were aware of their HIV status (41%) compared to those who were not aware of their HIV status (27%) (p-value = 0.000). Furthermore, in HIV-positive women, contraceptive prevalence was higher in those enrolled on integrated services (on ART) (40%) than those not on ART (28%) (*p*-value = 0.002). Women with viral suppression had higher contraceptive prevalence than those with unsuppressed viral loads. Contraceptive prevalence appears comparable by ART duration (Table 3). Age, education, marital status, sexual activeness, number of sexual partners, and number of birth and pregnancy as reported in reproductive histories were potential confounders associated with contraceptive use. Birth history showed the strongest association, where having a child born in the past five years had the highest modern contraceptive prevalence (50%). In HIV-negative women, similar confounders were observed, with the addition of employment status and social-economic status in the past seven days (Table 3).

**Table 3:** Prevalence of modern contraceptive use among HIV-positive and negative women by selected social, demographic, sexual, reproductive and HIV characteristics (*n* = 13,293)

Characteristic	HIV-positive		HIV-negative	
	**n (%)**	***P*-value**	**n (%)**	***P*-value**
All women	363 (35.7)		3643 (30.1)	0.004
**Enrolled on ART (Integration)**				
Yes	231 (40.1)	< 0.001		
No	112 (27.7)			
**Viral load suppressed (< 1000copies / mL)**				
Yes	229 (40.7)	0.004		
No	134 (29.4)			
**Duration of ART use (years)**				
1–2	69 (47.8)	0.263		
3–5	62 (35.7)			
6 +	58 (42.3)			
**HIV status awareness**				
Positive and aware	255 (41.1)	< 0.001		
Positive and unaware	108 (27.4)			
**Age categories**				
15–24	47 (32.0)	0.021	1095 (20.9)	< 0.001
25–34	143 (43.3)		1514 (42.2)	
35–49	173 (31.9)		1034 (31.4)	
**Education level**				
No formal education	46 (24.2)	0.005	(470) 26.1	< 0.001
Primary education	273 (39.5)		(2430) 33.6	
Secondary +	44 (29.9)		(739) 24.2	
**Residence**				
Urban	172 (35.1)	0.758	1369 (31.2)	0.091
Rural	191 (36.3)		2274 (29.4)	
**Currently working and receiving a regular salary**				
Yes	77 (33.5)	0.319	701 (36.0)	< 0.001
No	79 (41.5)		644 (33.2)	
Never worked in the past 12 months	207(34.5)		2296(27.9)	
**Household wealth**				
Low	199 (35.1)	0.7685	2193 (29.5)	< 0.001
High	164 (36.3)		1448 (30.8)	
**Marital status**				
Never married	34 (22.2)	< 0.001	399 (12.2)	< 0.001
Married/living with a partner	222 (43.4)		2780 (38.8)	
Divorced/Widow/Separated	107 (29.5)		464 (31.7)	
**Living arrangement**				
Living with partner	198 (43.8)	0.698	2587 (39.7)	0.001
Living apart	24 (40.5)		193 (31.2)	
**Currently sexually active**				
Yes	305 (40.2)	0.006	3146 (37.0)	< 0.001
No	57 (27.4)		452 (26.1)	
**Number of sexual partners**				
None	57 (27.4)	0.002	453 (26.1)	< 0.001
Only one	257 (43.0)		2787 (37.3)	
More than one	48 (30.7)		333 (36.7)	
**Number of previous pregnancies**				
None	6 (8.1)	< 0.001	174 (6.4)	< 0.001
1–2	98 (31.5)		1284 (38.3)	
3 +	259 (41.6)		2180 (38.5)	
**Number of children born in the past five years**				
0	160 (31.4)	< 0.001	914 (33.9)	< 0.001
1	150 (49.8)		1933 (45.0)	
2 +	36 (42.7)		543 (32.7)	
Never	6 (8.1)		174 (6.4)	

**Fig. 1:**
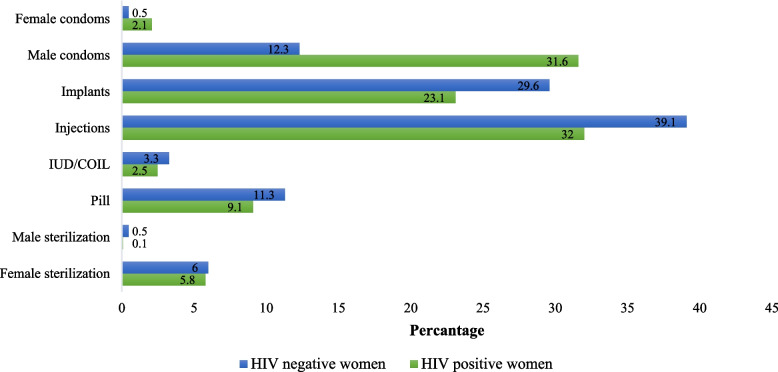
Types of modern contraceptives methods used by study participants (*n* = 4006)

The analysis of specific modern contraceptive methods used by HIV status shows injectables were the most common contraceptive option for both HIV-positive (32%) and HIV-negative women (39%). Implants were more common among HIV-negative (30%) than HIV-positive (23%). HIV-positive women reported the highest male condom use (31%) relative to HIV-negative women (12%). Both groups have similarities in using IUDs, pills, and sterilization. Both groups mostly preferred short-acting contraceptives, 69% (237) and 61% (2288), by HIV-positive and HIV-negative women, respectively (Fig. 1).

### Factors associated with modern contraceptive use among study participants (*n* = 13,293)

In the multivariate analysis combining HIV-positive and HIV-negative women (Model 1), women who knew their HIV-positive status had higher odds of modern contraceptives (AOR = 0.67, 95% CI:0.53–0.84) than HIV negtive women or HIV positive not knowing their status (Table 4). We adjusted Model 1 with nine potential confounders, of which six were associated with modern contraceptive use; age, formal education, number of sexual partners, marriage, employment history and a history of birth or pregnancy.

**Table 4:** Factors associated with modern contraceptive use among All and HIV-positive women (*n* = 13,293)

Characteristic	Model 1: HIV-positive and HIV-negative		Model 2: HIV-positive	
	**COR (95%CI)**	**AOR (95% CI)**	**COR (95%CI)**	**AOR (95% CI)**
**HIV-status awareness**				
Negative/Positive and unaware	Reference	Reference		
Positive and aware	0.61(0.50–0.76) ***	0.67(0.53–0.84) ***		
**Currently on ART**				
No			Reference	Reference
Yes			1.74(1.23–2.48) ***	1.85(1.27–2.71) ***
**Age categories**				
15–24	Reference	Reference	Reference	Reference
25–34	1.00(0.89–1.12)	1.02(0.88–1.19)	1.62(0.96–2.73)	0.86(0.47–1.56)
35–49	0.53(0.42–0.66)	0.57(0.48–0.67) ***	0.99(0.60–1.64)	0.47(0.25–0.88) **
**Education level**				
No formal education	Reference	Reference	Reference	Reference
Primary education	1.47(1.28–1.70) ***	1.70(1.46–1.98) ***	2.05(1.28–3.29) ***	2.03(1.22–3.37) ***
Secondary +	0.92(0.78–1.08)	1.71(1.41–2.09) ***	1.33(0.71–2.52)	1.60(0.80–3.23)
**Marital status**				
Never married	Reference	Reference	Reference	Reference
Married/living with partner	4.51(3.93–5.18) ***	1.29(1.07–1.55) ***	2.69(1.54–4.70) ***	1.39(0.71–2.74)
Divorced/Widow/Sep	3.21(2.68–3.83) ***	0.99(0.80–1.23)	1.47(0.82–2.64)	0.89(0.45–1.79)
**Number of sexual partners**				
None	Reference	Reference	Reference	Reference
only one	1.72(1.49–1.98) ***	1.63(1.41–1.88) ***	2.00(1.29–3.08) ***	1.79(1.12–2.86) ***
more than one	1.61(1.31–1.97) ***	1.75(1.42–2.16) ***	1.17(0.66–2.09)	1.43(0.75–2.74)
**Number of pregnancies**				
None	Reference	Reference	Reference	Reference
1–2	8.81(7.19–10.78) ***	3.62(2.88–4.54) ***	1.65(0.64–2.65) ***	3.43(1.26–9.33) ***
3 +	9.17(7.55–11.15) ***	5.15(3.97–6.64) ***	2.01(1.10–3.07) ***	6.52(2.47–17.18) ***
**Residence**				
Urban	Reference	Reference		
Rural	0.92(0.84–1.01)	0.89(0.77–1.02)		
**Household wealth**				
Low	Reference	Reference		
High	1.06(0.966–1.17)	1.08(0.94–1.23)		

COR-Crude odds ratio, AOR-Adjusted Odds Ratio

^**^Indicates *p*-value < 0.05

^***^ Indicates *p*-value < 0.001

In HIV-positive women (Model 2), enrolment into integrated services (currently on ART) demonstrated an increase in the odds of modern contraceptives by 85% (AOR = 1.85, 95%CI:1.27–2.71). Model 2 was adjusted for six variables, of which statistical significance were observed for age, formal education, number of sexual partners and number of pregnancies. HIV-positive women with only one sexual partner had 79% higher odds of modern contraceptive use compared to those with multiple partners (AOR = 1.79, 95%CI: 1.12–2.86). Additionally, those with more than three pregnancies had 6 times higher contraceptive use than those with fewer pregnancies (AOR = 6.52, 95%CI: 2.47–17.18); being aged 35 years and above, (AOR = 0.47, 95%CI: 0.25–0.88); and having primary education, (AOR = 2.03, 95%CI: 1.22–3.37) had significantly higher odds of modern contraceptive use (Table 4).

## Discussion

Tanzania adopted HIV and family planning services integration strategy to address unintended pregnancies and MTCT of HIV among HIV-positive women. HIV treatment and PMTCT clinics in Tanzania provide family planning counselling and prescribe or refer clients for contraceptive services [11, 12]. This study has assessed the impacts of enrollment into integrated services (ART) on modern contraceptive use among HIV-positive women in Tanzania. In a well-functioning integrated program, women enrolled on ART (integrated services) are likely to have higher modern contraceptives use than those not enrolled. We assessed this hypothesis by comparing contraceptive prevalence among HIV-positive women enrolled on integrated services (ART) and those not enrolled (HIV-positive not on ART or HIV-negative).

We analyzed nationally representative HIV impact survey data. Women on ART represent a subpopulation of HIV-positive potentially benefiting from family planning service integration through routine screening of contraceptive use, prescription and referrals as stipulated in family planning and HIV care and treatment guidelines.

This analysis demonstrated that, enrollment into integrated services (ART) is associated with increased modern contraceptive use among HIV positive women. Contraceptive prevalence by HIV status shows that approximately one-third of HIV-positive women (35%) used modern contraceptives. The prevalence was higher among those on integrated services (ART) (40%) compared to those not on ART or unaware of their status (27%) and HIV-negative women (30%).This indicates a 13% higher contraceptive prevalence among HIV-positive women on integrated services compared to those not on ART and unaware of their HIV status, and a 10% higher prevalence than HIV-negative women. This increase may be attributed to the integration of family planning and HIV services and or reduced desire for more children following HIV diagnosis [21, 22]. Prior studies have demonstrated that service integration increases the uptake of modern contraceptives among HIV-positive women [14, 15, 20]. A systematic review study by Grant-Maidment and colleagues reported 8% higher contraceptive use in integrated than on non-integrated facilities [15]. The difference in contraceptive prevalence in women on ART and those not in ART in this study is smaller than the expected impact of service integration observed elsewhere. In a prospective study in Rwanda, modern contraceptive prevalence increased from 30 to 72% following the service integration intervention [23].

In our multivariate analysis that combined HIV-positive and negative women (Model 1), HIV-positive status awareness increased the odds of modern contraceptives by 33%. In HIV-positive women (Model 2), being on integrated service increased the odds of modern contraceptives by 85%. These findings are consistent with the results reported among HIV-positive women in Malawi [24]. The increase may be attributed to family planning, HIV-service integration, and the intention to limit fertility and to avoid MTCT of HIV. Lower pregnancies and birth rates among HIV-positive women compared to HIV-negative women in this study support this hypothesis. Age composition may also be essential in describing high modern contraceptives use among HIV-positive people. More than half of HIV-positive women are in their middle ages (35–49) and possibly have attained their optimum number of children leading them to opt for no further pregnancies. This contrasts HIV-negative women who are adolescents or youth at their peak fertility age. Furthermore, our study found a 79% increase in modern contraceptive use among HIV positive women with only one sexual partner as compared to those with multiple partners. The higher use of contraception among HIV-positive women with only one sexual partner may be indicative of a greater focus on family planning and safer sex practices within monogamous relationships. This finding underscores the importance of tailored interventions for different risk profiles within the HIV-positive population, with a specific emphasis on promoting contraceptive use in the context of multiple sexual partners to further reduce unintended pregnancies and HIV transmission risks.

This study presents a precise and reliable situation of modern contraceptive use among HIV-positive women and in the general population in Tanzania. Our findings are comparable with studies from sub-Saharan African countries where low modern contraceptive prevalence was observed. These studies are from Kenya (32%) [25], Northern Uganda 25% [26], and Ghana 18%, 15% and 21% in 2003, 2008 and 2014, respectively [17]. Some countries have successfully managed to scale up modern contraceptives to the general population, particularly HIV-positive women. Our estimate is lower than the comparative study conducted in Ethiopia, where modern contraceptive prevalence in HIV-positive women was 94% and 73% in HIV-negative women [27]. Other studies reported high modern contraceptive prevalence are from Uganda (69%) [28] and South Africa (89%) [29]. These observations may be linked to the comprehensive integration of sexual and reproductive health and ART/HIV services in these countries [23–25].

One facility-based study in Kilimanjaro, Tanzania, reported a modern contraceptive prevalence of 54% among HIV-positive women attending HIV care and treatment clinics (CTCs) [30]. The higher contraceptive prevalence observed at CTCs may be linked to selection bias because those attending CTCs may be a subset of health-conscious women or from high-performing facilities. This study is representative and population-based, therefore more reliable due to the robust survey design, which included a random selection of study areas and households from the general population in a multistage process. Consequently, this approach often results in better estimates than health facility-based surveys.

The differences in the distribution of preferred contraceptive methods may be associated with many factors, such as contraceptive availability, individual preferences, and perceived risks by providers and users. Concerning perceived risks, the National Guideline on Family Planning in Tanzania cautions providers when prescribing oral contraceptives and implants due to reported reduced contraceptive efficacy among HIV-positive women treated with some ARV [31]. High-risk monocomponent or fixed ARV dose combinations include efavirenz, nevirapine and ritonavir/ritonavir. The guideline also prohibits the prescription of IUDs to patients poorly responding to ARV and those with untreated chlamydia and/or gonorrhoea, which are very common in HIV-positive individuals. HIV-positive women encountering these limitations remain with the male condom as the only option [31]. 

Contraceptive choice limitations in the family planning guidelines for HIV-positive may have contributed to high injectables and male condom use. Injectables and male condoms (32% and 31%, respectively) were the most common methods of contraceptives among HIV-positive women. The order of preference is consistent with a similar study from Malawi, where injectables (20%) and male condoms (13%) were the most popular methods of contraception among HIV-positive women [8]. A comparable study from Tigray, Ethiopia shows a similar pattern, where injectables (71%) and male condoms (48%) were the most preferred contraception method among HIV-positive [32]. Facility-based study in Kilimanjaro among HIV-positive women shows injectables and male condoms are the most preferred methods, but male condoms were the most preferred (76%), followed by injectables (28%) [33]. Similar observations have been reported in Uganda, where condoms were the most used method (61%) [28]. In HIV-negative women, this study shows injectables (39%) and implants (29%) were the most preferred method of contraception. A similar pattern was observed in the general population for injectables and male condoms, with an estimated contraceptive prevalence of 36% and 32%, respectively, in Tigray study [34]. An analysis of sexually active unmarried women in the general population in Tanzania shows injectables and male condoms were the most used methods of contraception (15% prevalence each) [35].

### Study limitations

The family planning and fertility modules of THIS 2016/17 survey collected data on a few variables, thus missing potential variables such as family planning knowledge, fertility intentions, male involvement, uptake of family planning counselling, and the sources of family planning information. The missing variables could have provided additional and valuable information for assessing the determinants of modern contraceptives among HIV-positive women. Morever, majority of HIV positive women (94%) were on ART, hence we can not make a definitive conclusion on weather being on ART was the only factor responsible for the observed difference in contraceptive use between women on ART and those not on ART.

## Conclusion

Despite the availability of the national guidelines for integrating family planning and HIV services, our study found lower modern contraceptive use among HIV-positive women in Tanzania than anticipated following integration. This suggests a gap in program effectiveness. As previously described, family planning is a cost-effective intervention for reducing unintended pregnancies and MTCT of HIV. These findings provide insight into why unintended pregnancies and MTCT of HIV persist in Tanzania. Therefore, we recommend a thorough evaluation of the family planning and HIV services integration program to identify factors that hinder or enhance its effectiveness in Tanzania.

## Data Availability

The study datasets are available from the THIS website through: https://phia.icap.columbia.edu/

## Appendix 1

Selected sexual, reproductive and HIV characteristics according to self-awareness of HIV status

**Table:**

Characteristic	HIV-positive (1066)		HIV-negative (13,830)	*P*-value
	**Self-aware (655)**	**Unaware (411)**		
**Currently sexually active**				
Yes	483(74.2)	324(81.6)	10,040(83.9)	
No	156(25.8)	76(18.4)	1939(16.1)	0.00
**Number of sexual partners**				
None	156(25.8)	76(18.5)	1967(16.4)	
only one	395(60.3)	246(60.7)	8887(74.2)	
more than one	86(13.9)	76(20.7)	1026(9.4)	0.00
**Relationship with last sex partner**				
Spouse/live in partner	254(61.2)	161(56.9)	6769(70.8)	
Non-cohabiting	165(38.8)	112(43.1)	2423(29.2)	0.00
**Condom use during last sex with a non-cohabiting partner**				
Yes	84(43.4)	37(20.0)	798(26.4)	
No	117(56.6)	97(80.0)	2112(73.6)	0.00
**Current pregnancy status**				
Pregnant	49(7.7)	27(6.2)	1248(11.6)	
Not pregnant	577(92.3)	337(93.8)	9411(88.4)	0.00
**Number of pregnancies, including a current pregnancy**				
None	27(5.1)	38(10.2)	2939(24.1)	
1–2	198(32.2)	124(32.8)	4066(30.3)	
3 +	430(62.6)	246(57.0)	6720(45.6)	0.00
**Number of children**				
0	324(52.0)	193(50.8)	3173(24.2)	
1	224(35.6)	112(29.1)	5032(36.9)	
2 +	47(6.9)	43(9.3)	1951(25.3)	
Never had children	27(5.5)	38(10.8)	1951(13.6)	0.00
**HIV disclosure to the partner**				
Yes	339(72.5)	98(37.9)	5739(63.2)	
No	111(27.5)	159(62.1)	3196(36.8)	0.00
**HIV status of the partner**				
Positive	180(63.7)	7(8.0)	75(1.1)	
Negative	100(36.3)	84(92.0)	5339(98.9)	0.00
**Suppressed viral load (< 1000 copies/mL)**				
Yes	513(79.3)	75(19.5)		
No	142(20.7)	336(80.5)		0.00
**Currently on ART**				
Yes	560(93.9)	48(12.7)		
No	42(6.1)	355(87.3)		0.00

## Abbreviations

AIDS: Acquired immune deficiency syndrome
AOR: Adjusted odd ratio
ANC: Antenatal Care
ART: Antiretroviral treatment
ARV: Antiretroviral drug
CI: Confidence interval
COR: Crude odd ratio
FP: Family planning
HIV: Human immunodeficiency virus
IUCD: Intra-uterine contraceptive devices
PMTCT: Prevention of mother-to-child transmission
SD: Standard deviation
THIS: Tanzania HIV impact survey
WHO: World health organization
WLHIV: Women living with HIV
